# Correction: Chemopreventive Efficacy of *Andrographis paniculata* on Azoxymethane-Induced Aberrant Colon Crypt Foci *In Vivo*

**DOI:** 10.1371/journal.pone.0119354

**Published:** 2015-04-07

**Authors:** The PLOS ONE Staff

The fourth author’s name is spelled incorrectly. The correct name is: Walaa Najm. The seventh author’s name is also listed incorrectly. The correct name is: Mahmood Ameen Abdulla*. Additionally, the second author’s name is listed incorrectly in the citation. The complete, correct citation is: Al-Henhena N, Poh R, Ismail S, Najm W, Khalifa SAM, El-Seedi H et al. (2014) Chemopreventive Efficacy of *Andrographis paniculata* on Azoxymethane-Induced Aberrant Colon Crypt Foci *In Vivo*. PLoS ONE 9(11): e111118. doi:10.1371/journal.pone.0111118

The section titled “Revised Results” should be titled “Results.”
